# Supplementary material for: Breathable, wearable skin analyzer for reliable long-term monitoring of skin barrier function and individual environmental health impacts
Source: Nat Commun. 2025 Oct 15;16:9149. doi: 10.1038/s41467-025-64207-2 (PMC12528383; doi:10.1038/s41467-025-64207-2)
Supplement: Supplementary file 1 — Supplementary Information [file 41467_2025_64207_MOESM1_ESM.pdf]

# Table of contents:

- **Supplementary Figures**


- Supplementary Figure 1. Device dimensions.
- Supplementary Figure 2. Image of web server for data collecting
- Supplementary Figure 3. Fabrication method of the BSA sensor.
- Supplementary Figure 4. Circuit design and board layout of the SH sensor.
- Supplementary Figure 5. Elimination of temperature fluctuations using NTCs under various conditions.
- Supplementary Figure 6. Temperature changes according to SH level in top and cross-sectional views.
- Supplementary Figure 7. Measurement Principle and Performance Analysis of the Skin Hydration (SH) Sensor.
- Supplementary Figure 8. Experimental and simulation result of chamber deforming under pressure.
- Supplementary Figure 9. Optimized variable stiffness of breathable chamber.
- Supplementary Figure 10. Schematics illustration of the actuator fabrication procedures.
- Supplementary Figure 11. Illustration of the SMA-based bistable actuator.
- Supplementary Figure 12. Finite-element simulation of the sensor module on skin under normal
- Supplementary Figure 13. Board layout of the BSA.
- Supplementary Figure 14. In vivo test of SH measurement compared with a commercial sensor and BSA.
- Supplementary Figure 15. Measurement of the commercial SH sensor.
- Supplementary Figure 16. Non-breathable patch type SH sensor.
- Supplementary Figure 17. TEWL measurement using tape stripping method.
- Supplementary Figure 18. Comparison of TEWL measurement by three subjects using a commercial sensor and the BSA on the same site.
- Supplementary Figure 19. Three-day continuous monitoring of skin barrier function using the BSA in three AD patients and three healthy controls.
- Supplementary Figure 20. Image of commercial PM measurement device.
- Supplementary Figure 21. Data processing of skin hydration, TEWL, and particulate matter exposure.
- Supplementary Figure 22. Change in SCORAD index relative to PM exposure.
- Supplementary Figure 23. Time lag correlation between PM exposure and epidermis SH, dermis SH and TEWL.
- Supplementary Figure 24. Environmental heal impact assessment using BSA after intervention.

## - Supplementary Tables

- Supplementary Table 1. Performance comparison with commercial skin health measuring devices.
- Supplementary Table 2. Performance comparison with wearable electronics.
- Supplementary Table 3. Comparison of skin hydration measured by electrical and transient heat transfer methods.
- Supplementary Table 4. Comparison of TEWL measurement methods.
- Supplementary Table 5. Bill of Materials.
- Supplementary Table 6. Thermal properties of each material used for transient heat transfer simulation analysis.
- Supplementary Table 7. Confusion matrix of clustering.
- Supplementary Table 8. Evaluation metrics of clustering.
- Supplementary Table 9. Baseline characteristics of the participants enrolled in this study.

## - Supplementary Notes

- Supplementary Notes 1. Measurement principle and calibration of Skin hydration sensor.
- Supplementary Notes 2. Measurement principle and calibration of TEWL sensor.
- Supplementary Notes 3. Skin indentation deformation.
- Supplementary Notes 4. SMA stress modelling.

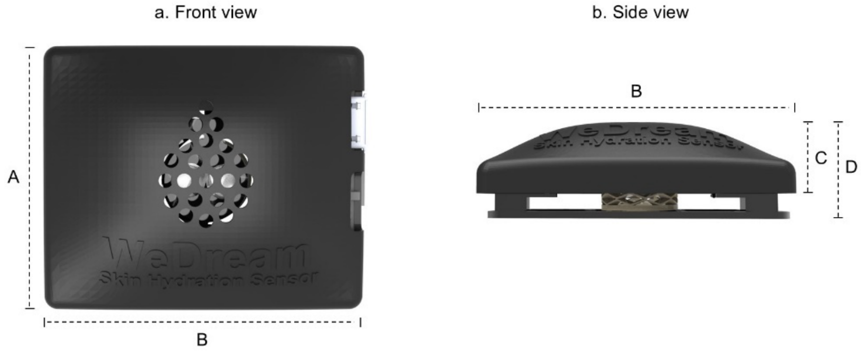


c. Device dimension in mm

| A | B | C | D |
|---|---|---|---|
| 38.5 | 46 | 10 | 15 |

**Supplementary Figure 1 | Device dimensions. a, Front view b, Side view and c, the values of the marked dimensions A, B, C and D in mm.**

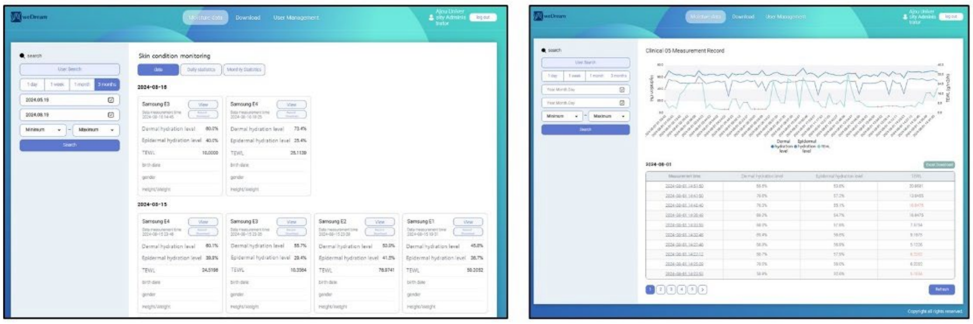

**Supplementary Figure 2 | Image of web server for data collecting.**

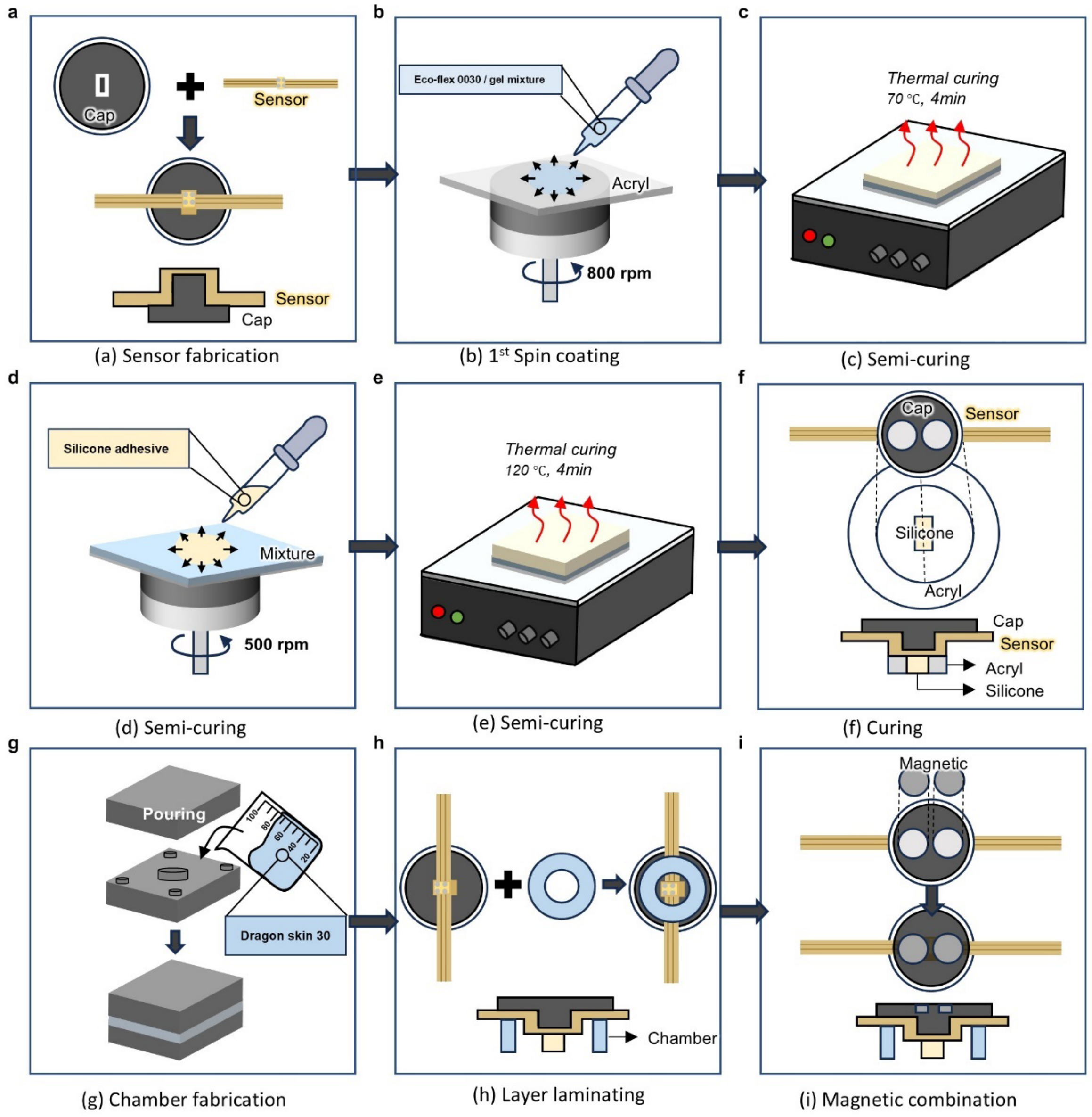


**Supplementary Figure 3 | Fabrication method of the BSA sensor.**

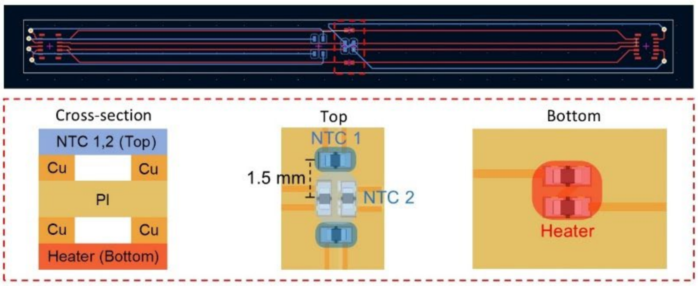


**Supplementary Figure 4 | Circuit design and board layout of the SH sensor.**

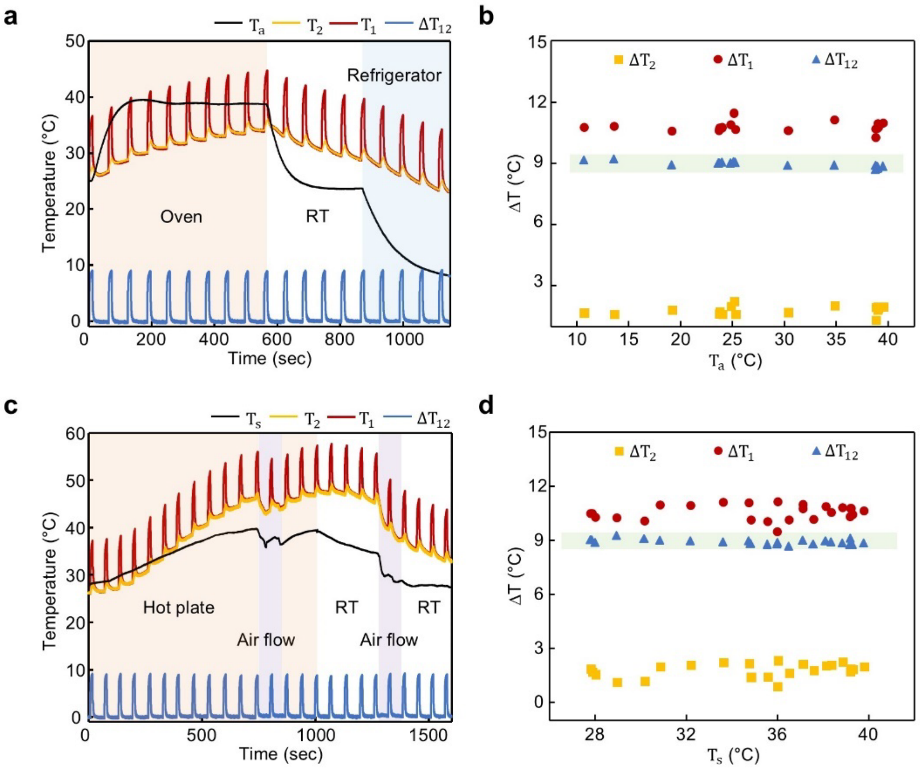


**Supplementary Figure 5 | Elimination of temperature fluctuations using NTCs under various conditions. a,** Measurements of $T_1$ (red), $T_2$ (yellow) and $\Delta T_{12}$ (blue) in various ambient temperature ($T_a$, black) using an oven and a refrigerator (red and blue background, respectively), **b,** Low sensitivity of $\Delta T_{12}$ to change in $T_a$ through temperature compensation ($\Delta T_1$-$\Delta T_2$). **c,** Measurements of $T_1$ (red), $T_2$ (yellow) and $\Delta T_{12}$ (blue) in various substrate temperature ($T_s$, black) using a hot plate and an air pump (red and purple background, respectively), **d,** Low sensitivity of $\Delta T_{12}$ to change in $T_s$ through temperature compensation.

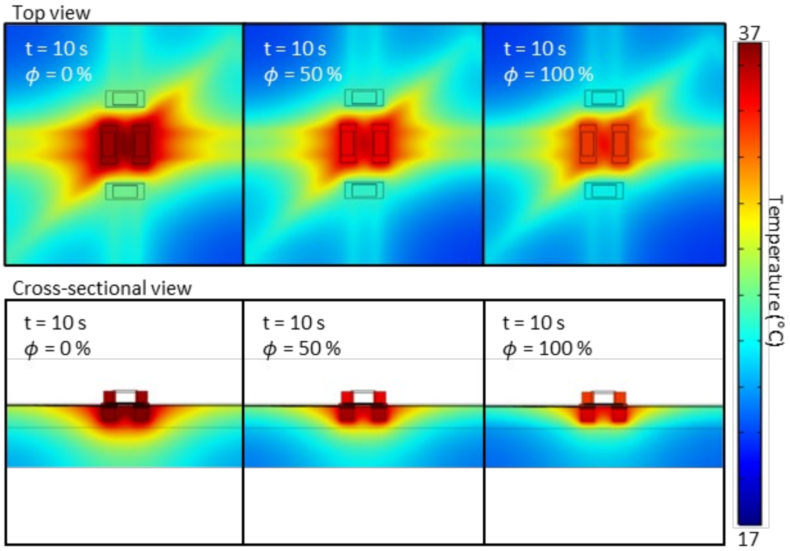


**Supplementary Figure 6 | Temperature changes according to SH level in top and cross-sectional views.** Temperature changes according to SH level using finite element method simulation

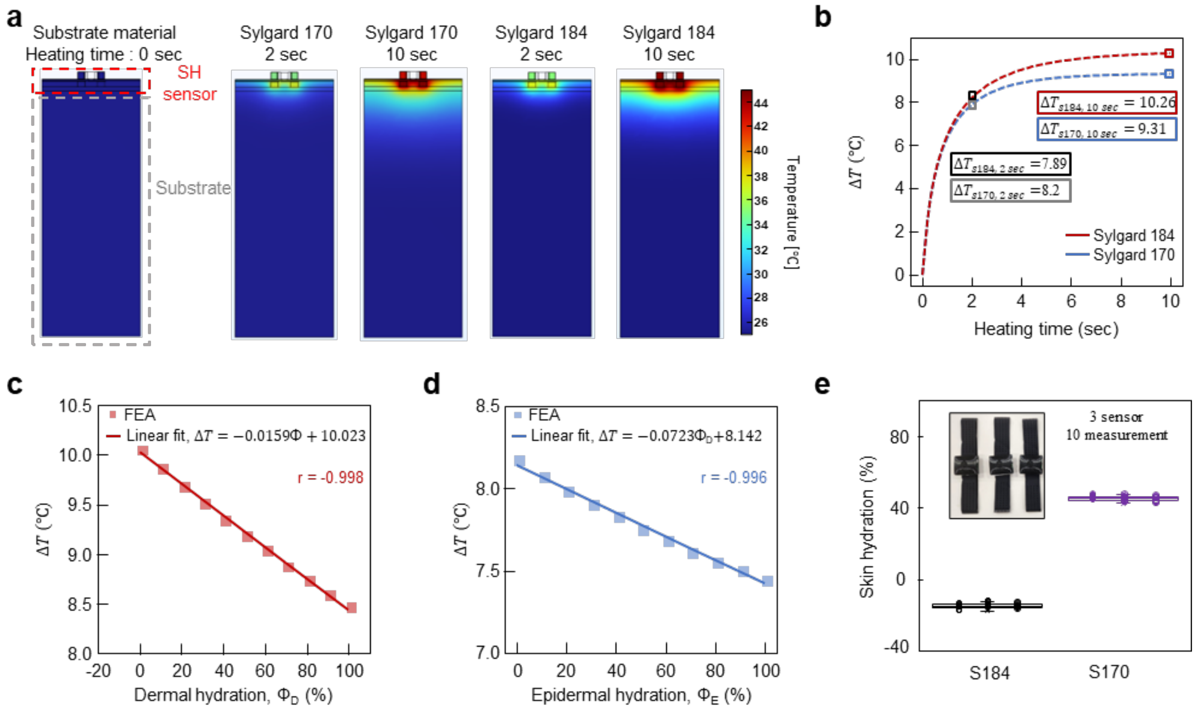


**Supplementary Figure 7 | Measurement Principle and Performance Analysis of the Skin Hydration (SH) Sensor. a,** Transient heat transfer simulation results at the epidermis (2 s) and dermis (10 s) for calibration materials (sylgard 170, sylgard 184). **b,** Correlation between heating time and the temperature difference measured by the NTC. **c,** Correlation between dermal hydration ($\Phi_D$) and NTC temperature difference with linear fitting results. **d,** Correlation between epidermal hydration ($\Phi_E$) and NTC temperature difference with linear fitting results. **e,** Measurement accuracy and repeatability of skin hydration based on calibration results. Measurements of SH for three BSA sensors are high accuracy and repeatability.

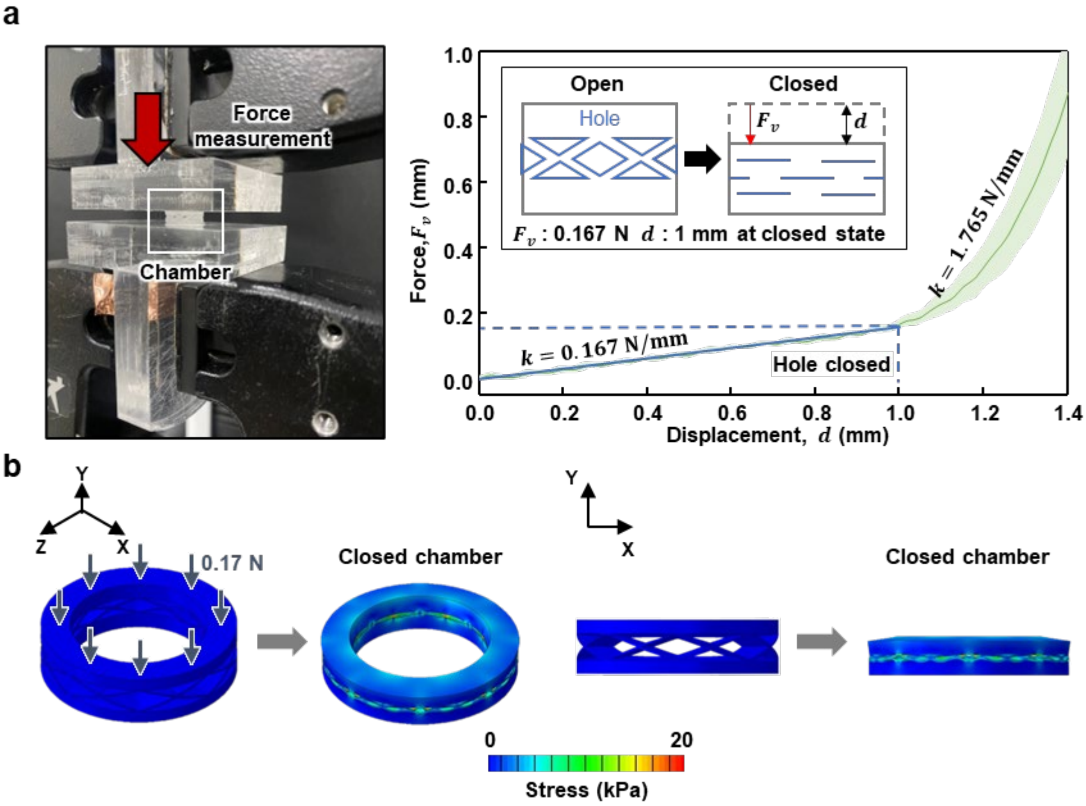


**Supplementary Figure 8 | Experimental and simulation result of chamber deforming under pressure. a,** Closing test of the breathable chamber. **b,** Simulation of the mechanical behavior of the breathable chamber.

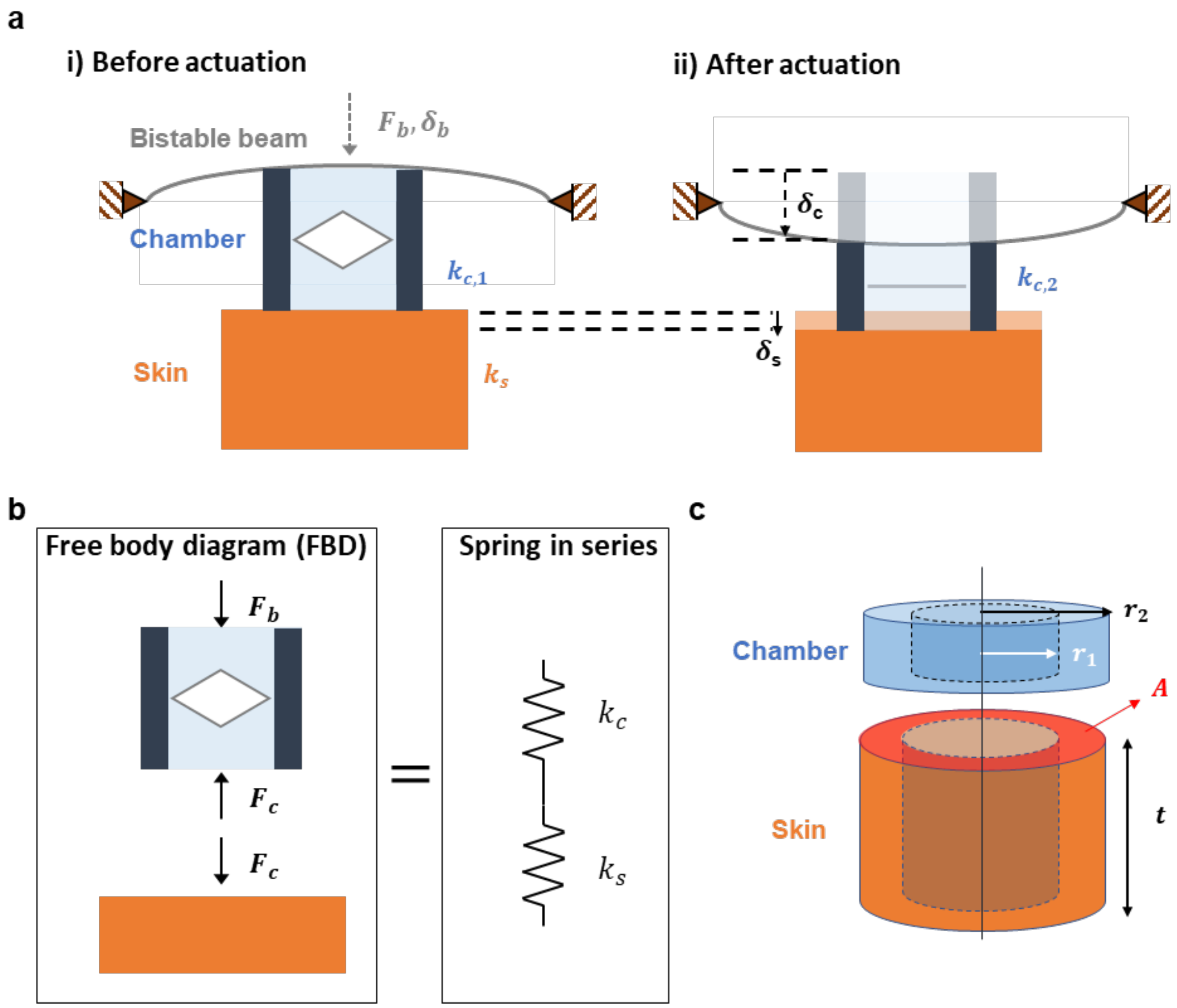


**Supplementary Figure 9 | Optimized variable stiffness of breathable chamber.** (a) Schematic illustration for mechanical behavior of actuator, breathable chamber, and skin. (b) Free body diagram of entire system and spring model. (c) Schematic for stiffness effective stiffness of skin.

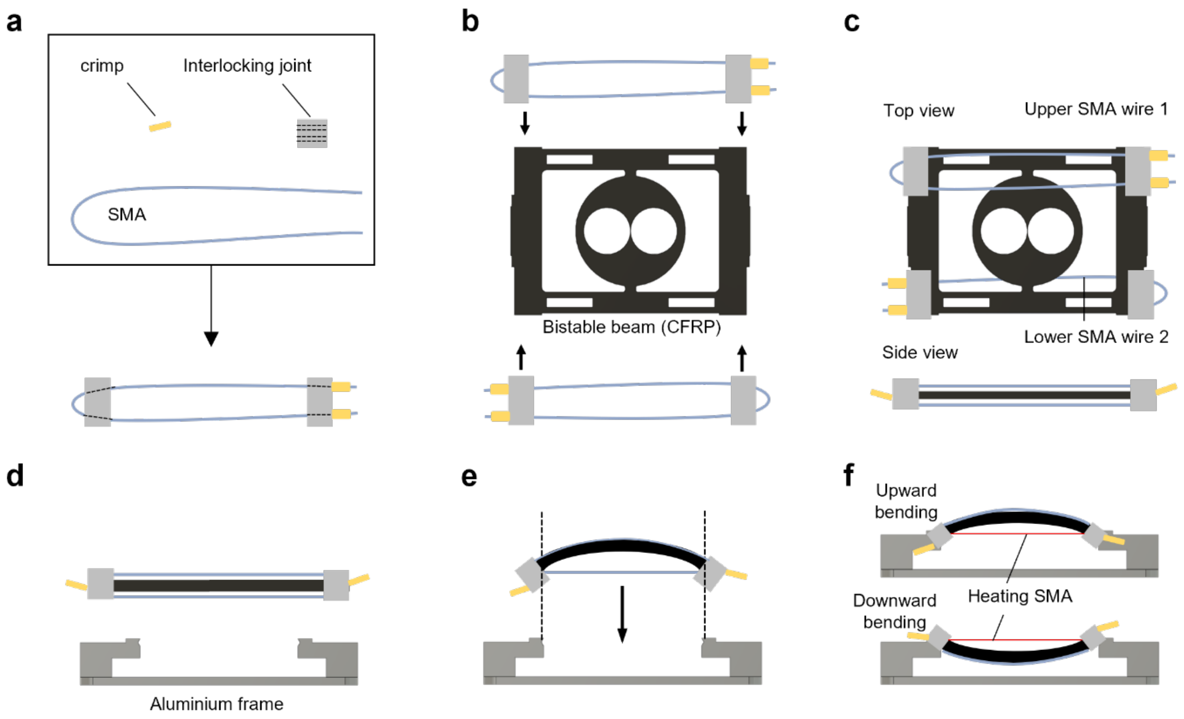


**Supplementary Figure 10 | Schematics illustration of the actuator fabrication procedures.**

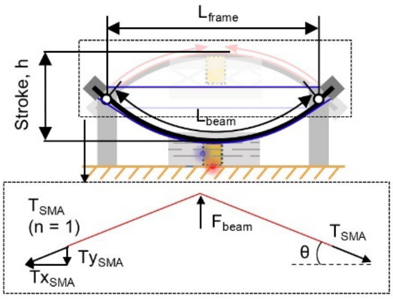


**Supplementary Figure 11 | Illustration of the Free body diagram of SMA wire.**

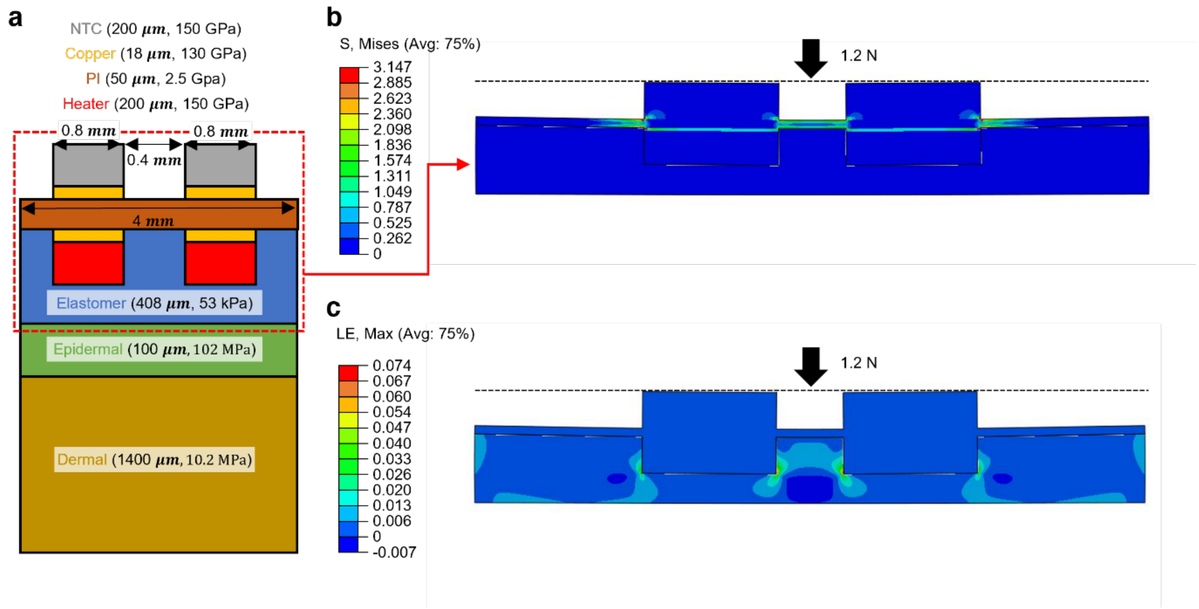


**Supplementary Figure 12 | Finite-element simulation of the sensor module on skin under normal a,** Schematic illustration of the sensor components and skin layers. **b,** Stress distribution in the sensor module subjected to a 12 N normal pressing load. **c,** Strain distribution under a 12 N normal pressing load.

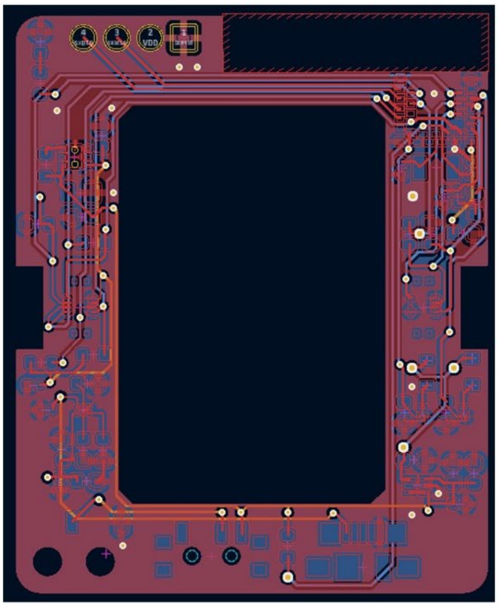

**Supplementary Figure 13 | Board layout of the BSA.**

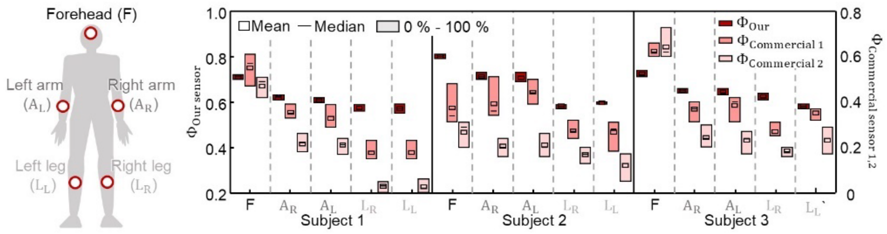


**Supplementary Figure 14 | In vivo test of SH measurement compared with a commercial sensor and BSA.**

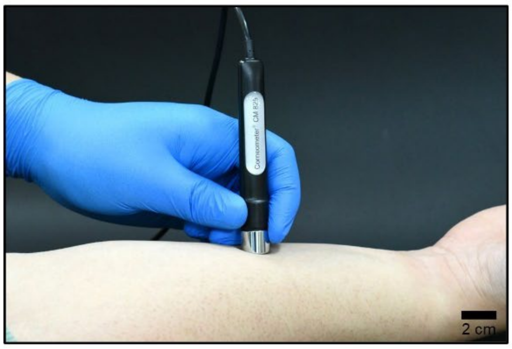


**Supplementary Figure 15 | Measurement of the commercial SH sensor.**

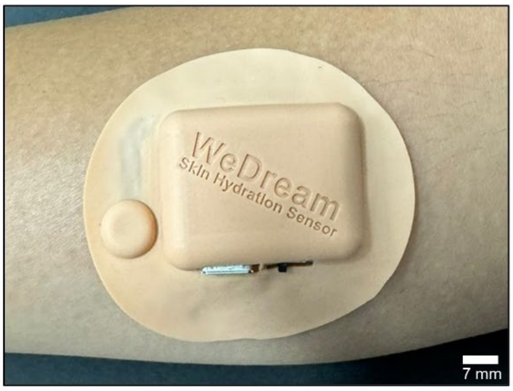


**Supplementary Figure 16 | Non-breathable patch type SH sensor.**

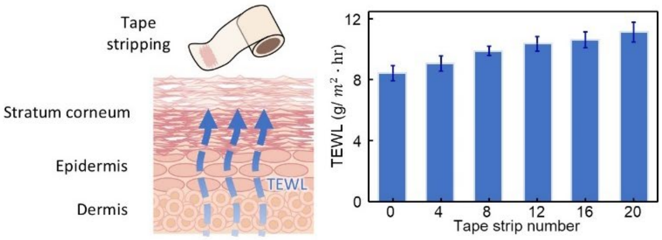


**Supplementary Figure 17 | TEWL measurement using tape stripping method.**

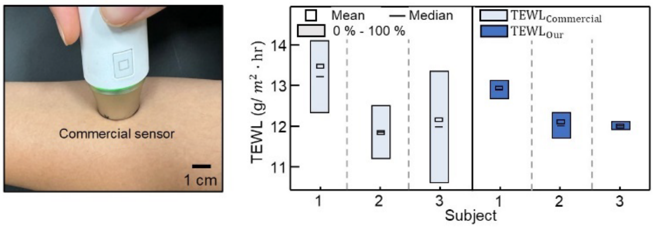


**Supplementary Figure 18 | Comparison of TEWL measurement by three subjects using a commercial sensor and the BSA on the same site.**

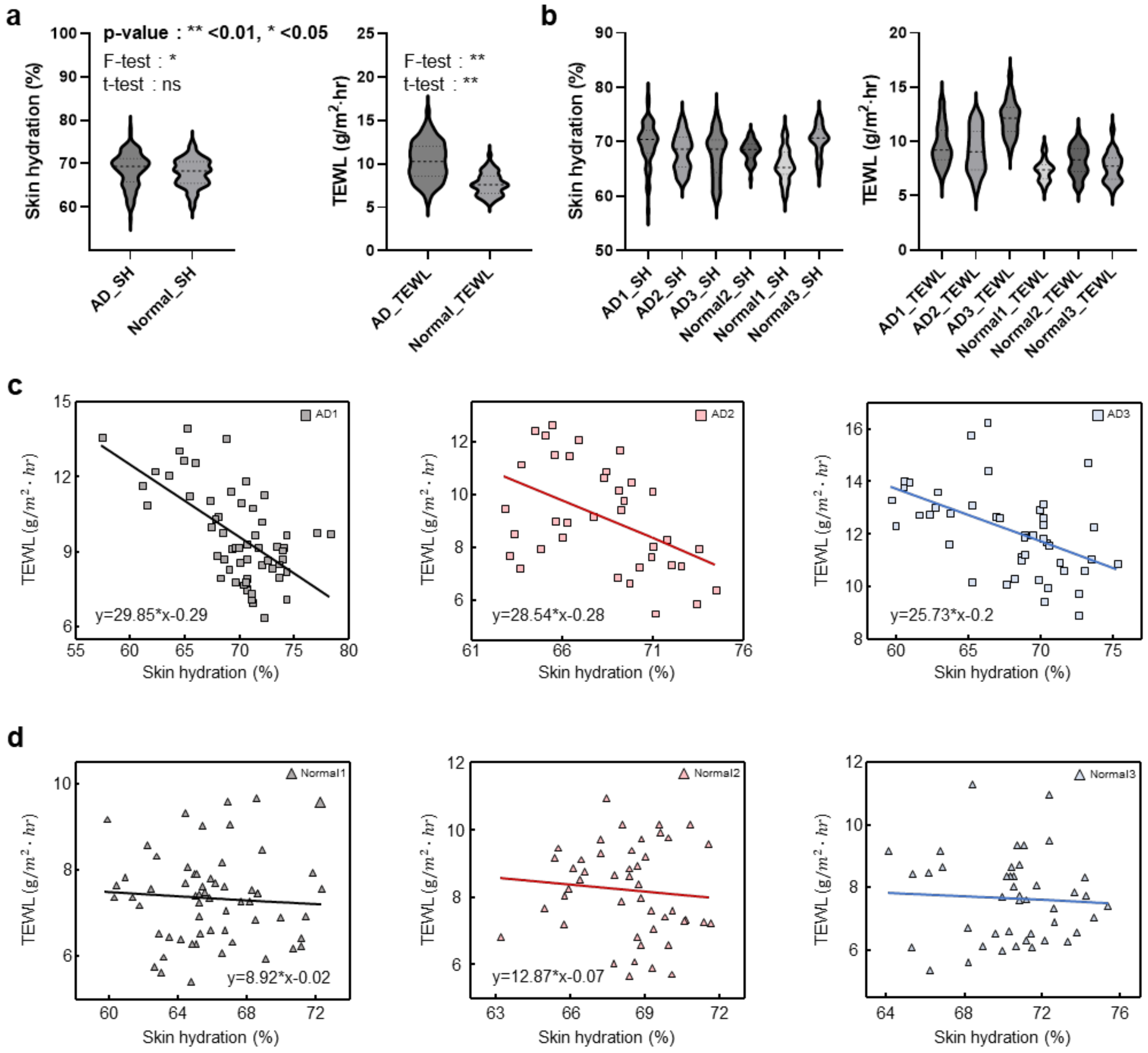


**Supplementary Fig. 19 | Three-day continuous monitoring of skin barrier function using the BSA in three AD patients and three healthy controls. a,** Violin plots of SH and TEWL data from 3 AD patients and 3 healthy controls with statistical comparisons using t-test (mean) and F-test (variance). **b,** Violin plots of SH and TEWL measurements from individual participants (AD1–3, Normal1–3), highlighting inter-individual variability within each group. Correlation between SH and TEWL for **c,** atopic dermatitis patients and **d,** normal subjects.

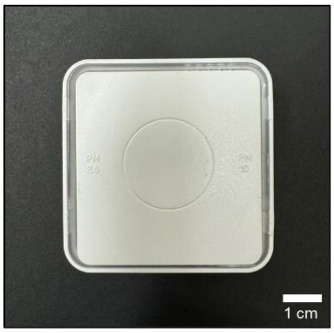


**Supplementary Figure 20 | Image of commercial PM measurement device.**

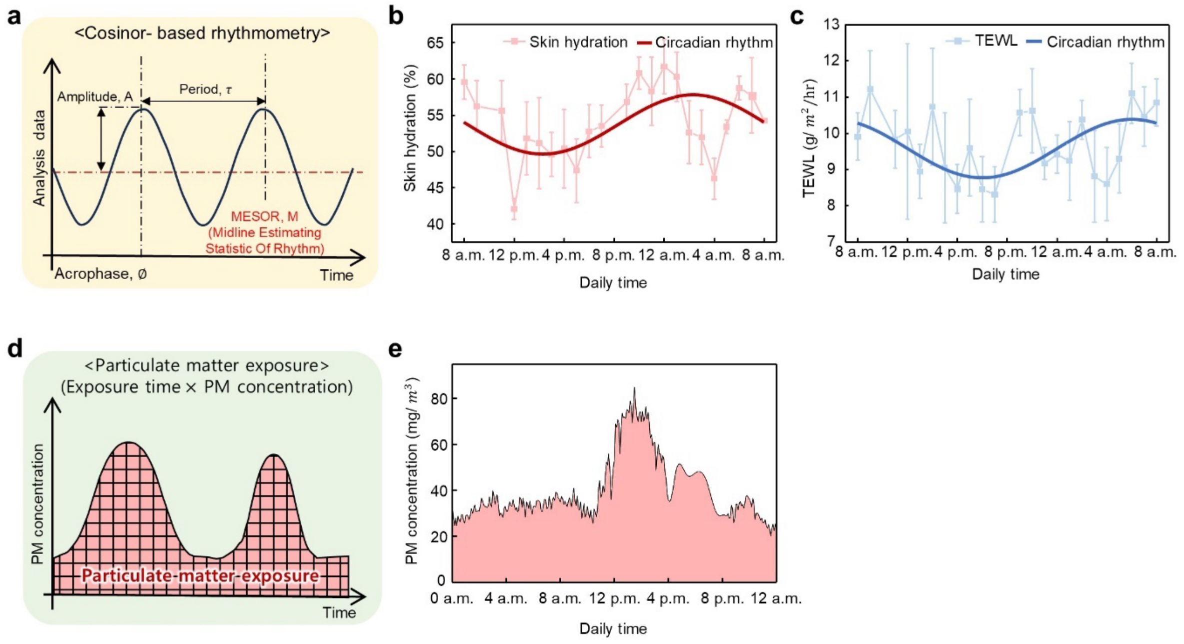


**Supplementary Figure 21 | Data processing of skin hydration, TEWL, and particulate matter exposure. a,** Schematic for cosinor-based rhythmometry. **b-c,** Circadian rhythm of skin hydration and TEWL. **d,** Schematic for particulate matter exposure calculation. **e,** Change in particulate matter concentration over daily time and calculation of particulate matter exposure.

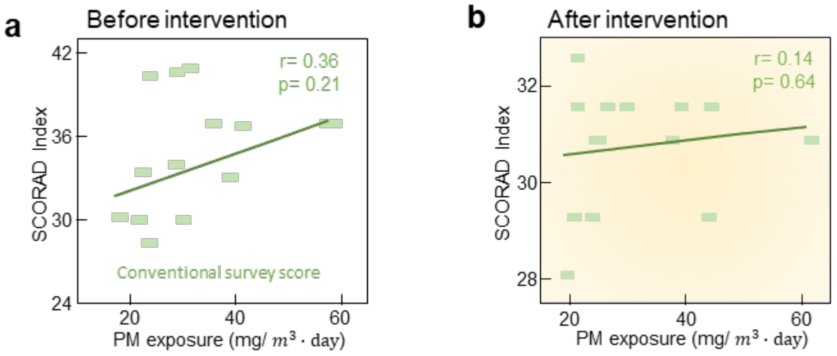


**Supplementary Figure 22 | Change in SCORAD index relative to PM exposure.**

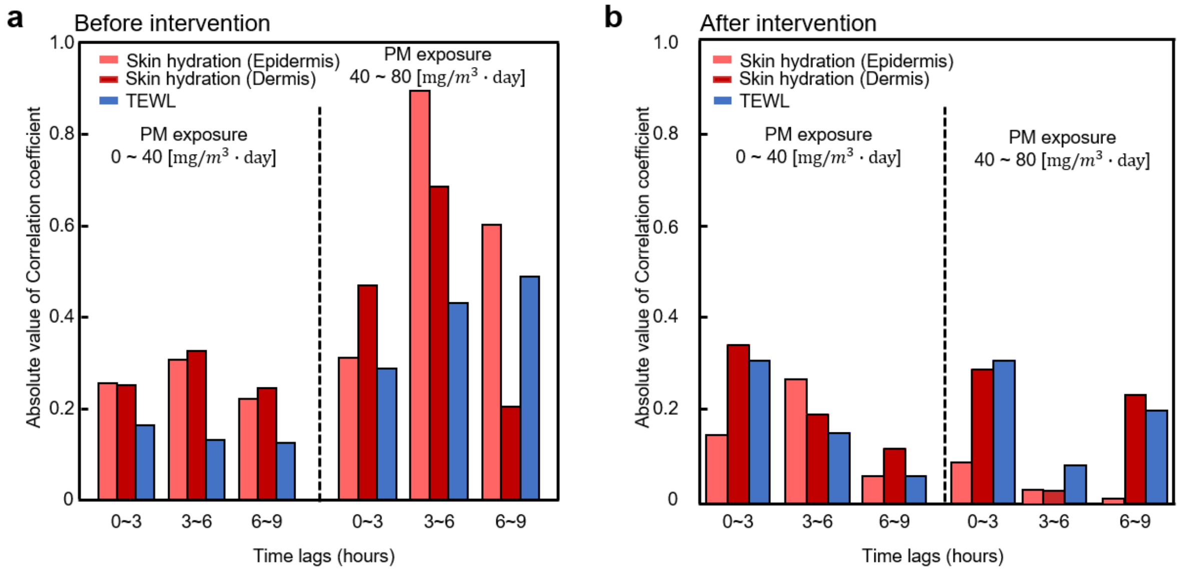


**Supplementary Figure 23 | Time lag correlation between PM exposure and epidermis SH, dermis SH and TEWL. a,** Bar graphs of correlation analysis between PM exposure and epidermis SH, dermis SH and TEWL, when before intervention PM exposure ranges from 0-40 and 40-80 mg/m$^3$day. **b,** Bar graphs of correlation analysis between PM exposure and epidermis SH, dermis SH and TEWL, when after intervention PM exposure ranges from 0-40 and 40-80 mg/m$^3$day. (The closer the value of the spearman correlation coefficient is to 1, the higher the correlation.)

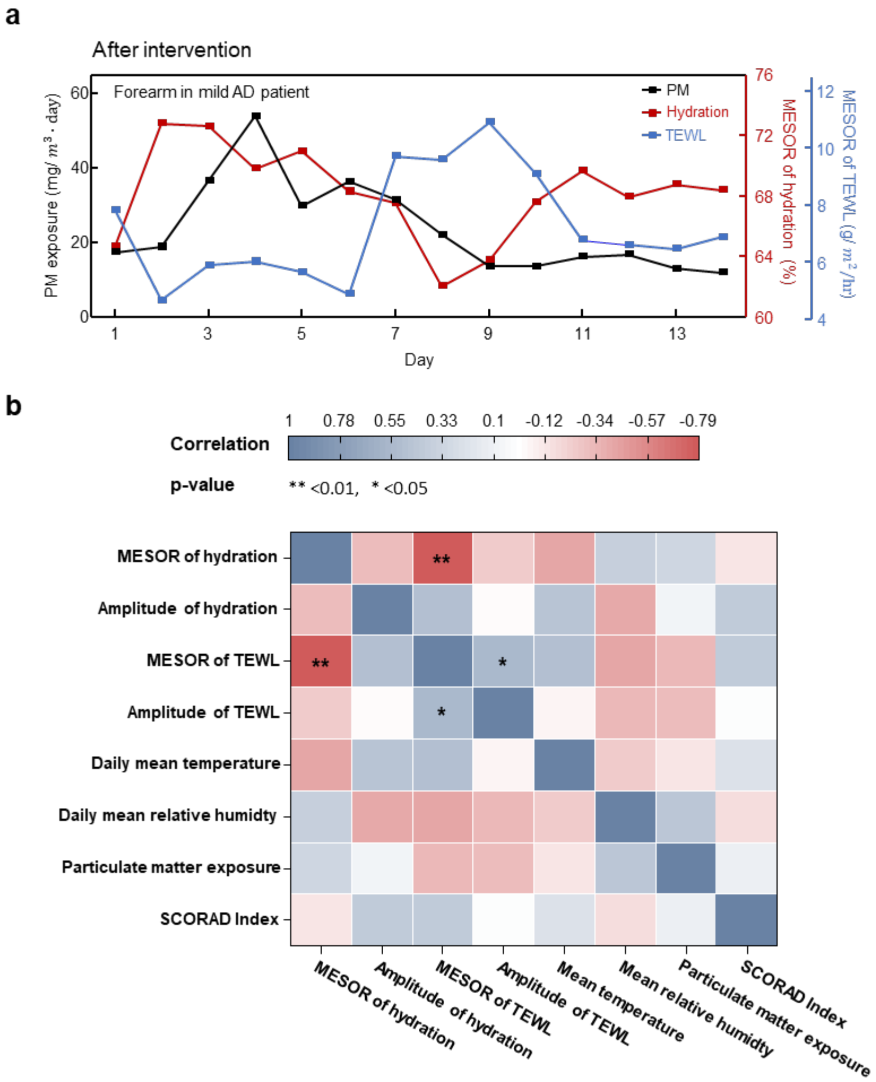


**Supplementary Figure 24 | Environmental heal impact assessment using BSA after intervention.**

| Ref | Commercial product | Corporation | Form factor | Measurement parameter | Measurement principle |
|---|---|---|---|---|---|
| 1 | Corneometer | GmbH | Stick | Skin hydration | Capacitance |
| 2 | Scalar | Scalar Corp | Stick | Skin hydration | Capacitance |
| 3 | DermaLab | CORTEX tech | Stick | Skin hydration<br>TEWL | Conductance<br>open chamber |
| 4 | Skicon | IBS | Stick | Skin hydration | Conductance |
| 5 | Nova DPM | Nova tech | Stick | Skin hydration | Impedance |
| 6 | GPskin barrier | Gpskin | Stick | Skin hydration<br>TEWL | Impedance<br>Closed chamber |
| 7 | Tewameter | GmbH | Stick | TEWL | Open chamber |
| 8 | Aquaflux | BioX | Stick | TEWL | Closed chamber |
| 9 | Epsilon | BioX | Stick | Skin hydration | Capacitance |

**Supplementary Table 1 | Performance comparison with commercial skin health measuring devices.** The Corneometer and Tewameter is golden standard of measuring skin hydration and TEWL respectively.

| Ref | Research Description | Disadvantages | Device Size | Breathability | Tethered / Untethered |
|---|---|---|---|---|---|
| Our Work | Heat transfer-based skin hydration measurement<br><br>Humidity sensor-based TEWL measurement<br><br>Chamber ventilation system for repetitive measurements<br><br>Bluetooth communication | - | ~38.5mm x 46mm | 98.6% | Untethered |
| [19] | Heat transfer-based skin hydration measurement | Low breathability<br><br>Not available for long term measurement | ~25mm x 40mm | - | Untethered |
| [22] | Highly breathable using nano-mesh electrodes<br><br>Capacitance based skin hydration measurement | Vulnerable to environment noise<br><br>Low durability | ~28mm x 20mm | - | Tethered |
| [24] | Highly breathable using micro holes<br><br>Capacitance based skin hydration measurement | Vulnerable to environment noise<br><br>Low durability | ~0.14mm x 0.22mm | 94.54% | Tethered |
| [18] | Heat transfer-based skin hydration measurement<br><br>Highly resistant to environment noise | Low breathability | ~0.9mm x 2.6mm | - | Untethered |
| [20] | Heat transfer-based skin hydration measurement<br><br>Highly resistant to environment noise | Low breathability | ~25mm x 35mm | - | Untethered |
| [27] | Skin attachable sensor<br><br>Capacitance based skin hydration measurement | Vulnerable to environment noise<br><br>Low durability<br><br>Low breathability | ~1.5mm x 1.5mm | - | Tethered |
| [26] | Conductivity based TEWL measurement<br><br>Highly breathable using nano-mesh electrodes | Vulnerable to environment noise<br><br>Low durability | ~14mm x 24mm (electrode)<br><br>16mm x 23mm (module) | - | Untethered |
| [33] | Humidity sensor-based TEWL measurement<br><br>Chamber ventilation system for repetitive measurements | Low wearability<br><br>Low breathability | 89.9mm x 40.9mm | - | Untethered |
| [30] | Humidity sensor-based TEWL measurement<br><br>Open chamber ventilation system | Vulnerable to environmental noise<br><br>Low wearability | 15mm x 15mm | - | Untethered |
| [29] | Resistance changes in the composite material based TEWL measurement<br><br>Highly breathable using hole pattern | Relatively low breathability than open chamber<br><br>Low wearability | 25mm x 30mm | 66.68% | Untethered |
| [31] | Humidity sensor-based TEWL measurement<br><br>Open chamber ventilation system | Vulnerable to environmental noise<br><br>Low wearability | ~ 80mm x 80mm | - | Untethered |

**Supplementary Table 2 | Performance Comparison with wearable electronics**

| Method | Conductance / Capacitance | Transient heat transfer |
| --- | --- | --- |
| Mechanism | 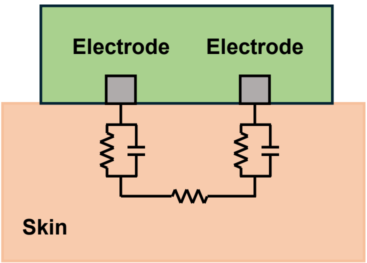  Apply DC/AC current to skin | 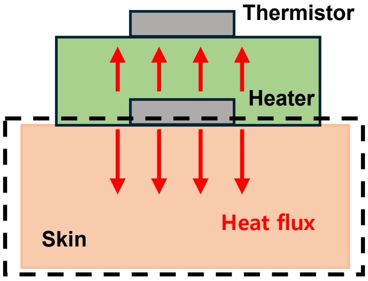  Thermal properties<br>(Thermal conductivity / diffusivity) |
| Advantage | Simple principle<br>Low measurement time (2 sec) | High accuracy<br>Measure dermis (deep skin) |
| Limitation | Low accuracy<br>Effect of cosmetics<br>Sensitive to pressure | High measurement time<br>(10 sec) |

**Supplementary Table 3 | Comparison of skin hydration measured by electrical and transient heat transfer methods.**

| Type | Open chamber method | Closed chamber method |
|---|---|---|
| Design | 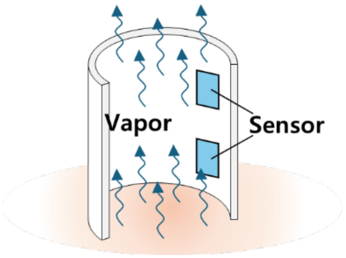  | 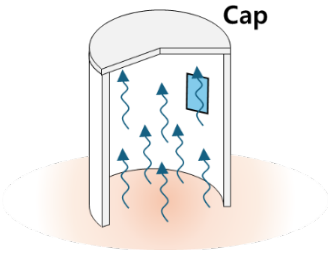  |
| Mechanism | Consists of two temperature and humidity sensors inside a hollow cylinder<br><br>Measure TEWL through humidity gradient | Inside a closed chamber, a sensor measures the rate of increase in relative humidity over a certain period of time and calculates TEWL. |
| Advantages | Simplicity<br><br>Does not disturb the skin<br><br>Continuous measurement possible | Less influenced by the external environment<br><br>Short measurement time |
| Disadvantages | Highly influenced by the external environment, such as disturbances due to movement of surrounding air | Ventilation is necessary because humidity continues to accumulate inside. |

**Supplementary Table 4 | Comparison of TEWL measurement methods.**

**a. Skin hydration & TEWL sensor part**

| Component | Quantity per sensor | Description | Manufacturer part number |
|---|---|---|---|
| NTC1+ / NTC-1 | 2 / 2 | THERMISTOR NTC 10KOHM 3380K 0201 | NCP03XH103J05RL |
| Heater | 2 | RES SMD 100 OHM 1% 1/10W 0603 | ERJ-3EKF1000V |
| SH1 | 1 | SENSOR HUMIDITY 100 RH SMD | SHT40-AD1B-R2 |
| CN1 | 2 | CONN PLUG 10POS SMD GOLD | 505274-1012 |

**b. Wireless communication & battery management system**

| Component | Quantity per sensor | Description | Manufacturer part number |
|---|---|---|---|
| C1 | 2 | CAP CER 12PF 50V C0G/NP0 0402 | GJM1555C1H120FB01D |
| C2 | 3 | CAP CER 10UF 6.3V X5R 0603 | CL10A106KQ8NNNC |
| C3 | 1 | CAP CER 1UF 50V X7R 0805 | CL21B105KBFNNNE |
| C4 | 4 | CAP CER 2.2UF 16V X5R 0603 | CL10A225KO8NNNC |
| C5 | 2 | CAP CER 0.1UF 25V X7R 0402 | CL05B104KA5NNNC |
| C6 | 1 | CAP CER 1UF 10V X7R 0603 | CL10B105KP8NNNC |
| C7 | 3 | CAP CER 1UF 6.3V X7R 0402 | CL05B105KQ5NQNC |
| D1 | 1 | LED BLUE CLEAR 0603 SMD | 150060BS75000 |
| L1 | 1 | FIXED IND 10UH 300MA 600MOHM SMD | MLZ1608N100LT000 |
| L2 | 1 | FIXED IND 15NH 460MA 0.16OHM SMD | LQW15AN15NJ00D |
| R1 | 9 | RES 10K OHM 1% 1/10W 0603 | RK73H1JTTD1002F |
| R2 | 1 | RES 1K OHM 1% 1/10W 0603 | RMCF0603FT1K00 |
| R3 | 1 | RES 6.04K OHM 1% 1/10W 0603 | RMCF0603FT6K04 |
| R4 | 1 | RES SMD 1.35KOHM 0.1% 1/10W 0603 | RT0603BRD071K35L |
| R5 | 1 | RES 0 OHM JUMPER 1/10W 0603 | RC0603JR-070RL |
| R6 | 2 | RES 4.22K OHM 1% 1/10W 0603 | RMCF0603FT4K22 |
| SW1 | 1 | SWITCH SLIDE SPDT 25MA 24V | EG1215AA |
| U1 | 1 | RF TXRX MOD BLUETOOTH CHIP SMD | MDBT42V-512KV2 |
| U2 | 1 | IC REG LINEAR 3.3V 150MA 6-WSON | TPS70933QDRVRQ1 |
| U3 | 1 | IC BATT CHG LI-ION 1CELL 6DSBGA | BQ25100YFPR |

| | | | |
|---|---|---|---|
| U4 | 1 | CONN RCPT USB2.0 MICRO B SMD R/A | 0473460001 |
| U5 | 2 | MOSFET N-CH 12V 2.9A 3PICOSTAR | CSD13383F4T |
| U6 | 1 | IC REG LINEAR 1V 300MA 6-WSON | TPS7A1010PDSET |
| X1 | 1 | CRYSTAL 32.7680KHZ 12.5PF SMD | ECS-.327-12.5-34B-TR |
| CN1 | 1 | CONN RCPT 10POS SMD GOLD | 5052701012 |

**Supplementary Table 5 | Bill of Materials. a,** Skin hydration & TEWL sensor part. **b,** Wireless communication & battery management system.

| **Material** | **Thermal conductivity, k (W/mK)** | **Thermal diffusivity, $\alpha$ ($m^2/s$)** | **Density, $\rho$ ($kg/m^3$)** | **Specific heat capacity, $C_P$ (J/kgK)** |
|---|---|---|---|---|
| Moist skin | 0.6 | 0.14 | - | - |
| Dry skin | 0.2 | 0.15 | - | - |
| Polyimide | 0.55 | - | 1340 | 3731 |
| Copper | 377 | - | 8940 | 385 |
| Skin adhesive | 0.164 | 0.12 | 969 | 1405 |
| Silver | 429 | - | 10500 | 234 |
| Alumina | 20 | - | 3900 | 900 |
| Sylgard 184 | 0.165 | 0.11 | 987 | 1509 |
| Sylgard 170 | 0.321 | 0.208 | 1327 | 1177 |

**Supplementary Table 6 | Thermal properties of each material used for transient heat transfer simulation analysis.** Specific heat capacity = thermal conductivity/density*thermal diffusivity [J/(kg·K)]

| | True | False |
|---|---|---|
| Positive | True-Positive (TP): 78 | False-Positive (FP): 2 |
| Negative | True-Negative (TN): 14 | False-Negative (FN): 4 |

**Supplementary Table 7 | Confusion matrix of clustering.**

| Evaluation metrics | Formulas | Value |
|---|---|---|
| **Rand measure index** | $RI = \frac{TP + TN}{TP + FP + FN + TN}$ | **0.94** |
| **Jaccard index** | $J(A, B) = \frac{TP}{TP + FP + FN}$ | **0.93** |
| **Fowlkes-Mallows index** | $FM = \sqrt{\frac{TP}{TP + FP} \times \frac{TP}{TP + FN}}$ | **0.96** |

**Supplementary Table 8 | Evaluation metrics of clustering.**

| Subject /Number | Age | Sex | Ethnicity | Pathology |
|---|---|---|---|---|
| **Normal 1** | **33** | **M** | **Asian** | **Healthy** |
| **Normal 2** | **29** | **F** | **Asian** | **Healthy** |
| **Normal 3** | **27** | **M** | **Asian** | **Healthy** |
| **AD 1** | **26** | **M** | **Asian** | **Atopic dermatitis** |
| **AD 2** | **29** | **F** | **Asian** | **Atopic dermatitis** |
| **AD 3`** | **24** | **M** | **Asian** | **Atopic dermatitis** |

**Supplementary Table 9 | Baseline characteristics of the participants enrolled in this study.**

*Supplementary Note 1. Measurement principle and calibration of Skin hydration sensor*

The skin hydration sensor in the breathable skin health analyzer consists of two Wheatstone bridges and one pair of heating resisters. Each Wheatstone bridge consists of two NTC thermistors and two 10-kohm resistors. The NTC thermistor on top of the resistor heating element is NTC 2, with NTC 1 located 1.5 mm away from NTC 2. (Supplementary Fig. 4) The resistance and temperature of the NTC thermistors have the relationship shown in Equation (1).

$$\ln\left(\frac{R_{NTC}}{R_0}\right) = \beta\left(\frac{1}{T} - \frac{1}{T_0}\right) \tag{1}$$

The potential difference of the nodes in each Wheatstone bridge satisfies Equation (2).

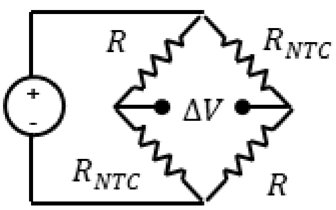


**Wheatstone bridge**

$$\Delta V = V_S\left(\frac{R}{R+R_{NTC}} - \frac{R_{NTC}}{R+R_{NTC}}\right) = V_S\left(\frac{R-R_{NTC}}{R+R_{NTC}}\right)$$

$$\Delta V(R + R_{NTC}) = V_S(R - R_{NTC}),\ \ R_{NTC}(\Delta V + V_S) = R(V_S - \Delta V) \tag{2}$$

$$\frac{R_{NTC}}{R} = \frac{V_S - \Delta V}{\Delta V + V_S}$$

$$\therefore\ ln\frac{R_T}{R_0} = ln\frac{R_{NTC}}{R} = ln\frac{V_S - \Delta V}{\Delta V + V_S} = \ln\left(\frac{2V_S}{\Delta V + V_S} - 1\right)$$

Using Equations (1) and (2), the relationship between the thermistor temperature T and the potential difference at the nodes can be expressed as Equation (3).

$$\frac{1}{T} = \frac{1}{\beta}\left[\ln\left(\frac{R}{R_0}\left(\frac{2V_S}{V_S + \Delta V} - 1\right)\right)\right] + \frac{1}{T_0} \tag{3}$$

To implement the skin moisture measurement mechanism, an analytical model of the variation of skin thermal conductivity $\boldsymbol{k}$ and thermal diffusivity $\boldsymbol{\alpha}$ with skin hydration was modeled by the Maxwell-Eucken model. According to the Maxwell-Eucken model, if moisture with a thermal conductivity of $\boldsymbol{k_w}$ is contained within dry skin with a thermal conductivity of $\boldsymbol{k_d}$and the water volume fraction is ϕ, the thermal conductivity of the skin, $\boldsymbol{k_{skin}}$, satisfies equation (4).

$$\frac{k_{skin}}{k_d} = 1 + \frac{3\phi}{\left(\frac{k_w + 2k_d}{k_w - k_d}\right) - \phi} \tag{4}$$

This expression can be written in terms of $\boldsymbol{k_{skin}}$, as Equation (5).

$$k_{skin} = \frac{(p+2) + 2(p-1)\phi}{(p+2) - (p-1)\phi} k_d, \qquad (p = \frac{k_w}{k_d}) \tag{5}$$

Based on the definition of thermal diffusivity, the thermal diffusivity $\boldsymbol{\alpha_{skin}}$ of a skin has the relationship shown in Equation (6).

$$\alpha_{skin} = \frac{k_{skin}}{\rho_{skin} c_{skin}} \tag{6}$$

Where $\boldsymbol{\rho_{skin}}$ is the density of the skin and $\boldsymbol{c_{skin}}$ is the isobaric specific heat capacity of the skin. To convert Equation (6) to equations for dry skin and moisture, the lumped parameter method was used. The lumped parameter method uses Equation (6) to calculate and predict the thermal diffusivity from the effective thermal conductivity, effective density, and effective isobaric specific heat capacity. According to the lumped parameter method, it has the relationship of Equation (7).

$$\rho_{skin} c_{skin} = \rho_d c_d (1 - \phi) + \rho_w c_w \phi \tag{7}$$

Substituting Equations (5) and (7) into Equation (6), the thermal diffusivity of the skin, α_skin, can be expressed as Equation (8).

$$a_{skin} = \frac{\alpha_w \alpha_d k_{skin}}{(1-\phi)\alpha_w k_d + \phi \alpha_d k_w} \tag{8}$$

To measure the skin hydration, we need to find the relationship between the thermal conductivity and thermal diffusivity of the skin according to the skin moisture (Equation (8)) and the temperature change of the thermistor (Equation (3)) obtained above. To find this relationship, we performed finite element analysis (FEA). The commercial program for finite element analysis was COMSOL Multiphysics® 5.6, and the analysis was performed with a 3D model. The physical phenomenon analyzed is heat transfer in solids. The entire region of the

model was set as a solid, and the governing equation for the solid region was set as Equation (9).

$$\rho c \frac{\partial T}{\partial t} + \rho c u \cdot \nabla T + \nabla \cdot q = Q + Q_{ted}$$
$$q = -k \nabla T \qquad (9)$$

$\boldsymbol{T}$ is the temperature, $\boldsymbol{t}$ is the time, $\boldsymbol{u}$ is the fluid velocity vector, $\boldsymbol{Q}$ is the energy of the heat source, and $\boldsymbol{Q_{ted}}$ is the energy of thermoelastic damping. The initial value for the entire model domain is set to 293.15 K, and the heat flux is set to the surface of the model. The material form of the heat flux is non-solid, and the heat flux phenomenon satisfies Equation (10).

$$q_0 = h \cdot (T_{ext} - T) \qquad (10)$$

$\boldsymbol{q_0}$ is the internal heat flux, $\boldsymbol{h}$ is the heat diffusion coefficient, and $\boldsymbol{T_{ext}}$ is the external temperature. We set the thermal diffusivity coefficient $\boldsymbol{h}$ to $\boldsymbol{25W/m^2K}$ and the external temperature $\boldsymbol{T_{ext}}$ to $\boldsymbol{293.15K}$. The heater part of the model is set as a heat source. The heat generation rate $\boldsymbol{P_0}$ of the heat source is set to $\boldsymbol{0.0352W}$. Skin hydration is measured using a transient heat transfer method, which involves three steps. First, skin temperatures at the heater location and at a point 1.5 mm away from the heater are measured using a Wheatstone bridge and an NTC thermistor. Based on the measured temperature distribution, the thermal properties of the skin (thermal conductivity and thermal diffusivity) are calculated using the finite element method (FEM). Finally, skin hydration is estimated from the obtained thermal properties using the Maxwell–Eucken model.

The principle of the calibration of skin hydration sensor is based on transient heat transfer, and the linear governing relationship between skin hydration ($\phi_s$) and the temperature difference ($\Delta$T) between two pairs of NTC thermistors was derived through finite element analysis (FEA) (Supplementary Fig. 7). Depending on the heating duration—2 seconds and 10

seconds—heat transfer into the skin to depths corresponding to the epidermis and dermis, respectively. The resulting temperature differences enable the estimation of epidermal hydration ($\phi_E$) and dermal hydration ($\phi_D$). Two materials (Sylgard 170 and Sylgard 184) were used as calibration standards. Their thermal properties correspond to 34.9% and –7.9% hydration levels in the epidermis, and 44.9% and –14.9% in the dermis, respectively. We can approximate the skin moisture as a function of temperature difference by modeling it with a first-order equation. (Equation (1), Fig.2d)

$$\Delta T = \mathrm{A} \times \phi + B \qquad (11)$$

Therefore, by measuring the temperature difference between the above two materials, we can obtain the coefficients A,B by combining Equations (12)

$$\begin{aligned} \Delta T_{S184} &= A \times \phi_{S184} + B \\ \Delta T_{S170} &= A \times \phi_{S170} + B \end{aligned} \qquad (12)$$

This equation can be applied to both epidermal and dermal moisture levels to obtain four calibration factors, two each. The calibration factors are obtained by measuring the temperature difference between the skin and a material with similar thermal properties so that the device can reliably provide a wide range of skin moisture values caused by skin diseases. Through sensor calibration, we were able to improve not only the performance of individual sensors but also ensure consistency across different sensors. The results showed high accuracy and repeatability. When the three sensors were tested 10 times, the error between the mean value and true value of each sensor was less than 1%, and the error within each sensor was less than 2%.

.

*Supplementary Note 2. Measurement principle and calibration of TEWL sensor*

The measurement of TEWL uses the closed chamber method (CCM), which consists of a closed sylinder with a relative humidity sensor[1,2]. The sensor measures collected water vapour from the skin in the chamber from which it cannot escape. The CCM provides a more stable environment for TEWL measurements than the open chamber method, because it reduces the influence of external environmental factors such as ambient airflows and minimizes skin damage by allowing for a shorter measurement time[3](Supplementary Table S4).

The calibration of the TEWL sensor was performed using the wet-cup method[4]. In this approach, a dish filled with water is covered with a semi-permeable membrane to establish a controlled surface from which water consistently evaporates. The mass change of the water is measured over time while maintaining a temperature, enabling the calculation of the evaporation rate per unit area (*g/m²/h*). The humidity gradient is directly related to the amount of water within the chamber[5], with the water quantity *m(t)* at time *t* determined as described by equation (4):

$$m(t) = m_0 + \Delta mtA = m_0 + (constnat)ktA \quad (4)$$

where $m_0$ is an initial water mass. Here, *Δm* represents the increase in hymidity per unit time and unit area, with units of *g/m²/h*, and A denotes the evaporation area. *Δm* is directly proportional to the slope *k* as described by equation (5):

$$\Delta m = (constant)\ k \quad (5)$$

The proportionality constant (*k*) can be easily determined by measuring the reduction in the evaporated water mass. The calculated $\Delta m$ represents the TEWL and serves as the reference data for the calibration of the TEWL sensor in the BSA. To calibrate the sensor, we measured the humidity change over time in the same wet-cup method setup as the reference

data, derived $k_2$, and then determined the calibration factor (CF) to match with the previously obtained reference data as shown in equation (6):

$$m = \text{TEWL} = (constant)k = k_2 \times CF \tag{6}$$

Additionally, to precisely measurement of TEWL, which exhibits a wide range in atopic patients, we compared the CF derived from measurements (*N* = 5) of evaporation from a single layer of a semi-permeable membrane under two conditions using wet-cup method: using water at room temperature (23°C) and water at 40°C. The TEWL for water at room temperature was 5.1±0.4 $g/m^2/h$, and for water at 40°C, it was 23.7±0.9 $g/m^2/h$. The corresponding *k* values were 0.21 and 0.98, respectively, resulting in a CF of approximately 24 for the TEWL sensor of the device.

*Supplementary Note 3. Skin indentation deformation*

To accurately measure TEWL, it is crucial that the breathable chamber maintains a constant volume even after closing. The chamber features side holes for high breathability and has a variable stiffness characteristic, exhibiting low stiffness ($k_{c,1}$) before the holes close and higher stiffness ($k_{c,2}$) after closing. The variable stiffness can be defined as described in Equation (13) by analyzing the force-displacement curve of the breathable chamber. When the chamber on the skin is pressed with a force $F_b$ by an actuator, a displacement $\delta_b$, which in turn causes the displacements $\delta_c$ in the chamber and $\delta_s$ in the skin, as expressed by equation (14) (Supplementary Fig. 8a). By solving the equilibrium equation from the free body diagram using Equation (15), it is shown that $F_b$ and $F_c$ are equal, as demonstrated in Equation (16) (Supplementary Fig. 8b). By substituting it into Equation (14), the deformation of the entire system behaves according to the spring series model as described in Equation (17), where the displacements of the chamber and the skin are inversely proportional to their stiffness coefficients (Supplementary Fig. 8b right). Therefore, to maintain a consistent chamber volume for reliable measurements, the stiffness coefficient of the chamber before closing ($k_{c,1}$ = 0.167 N/mm) is smaller than the stiffness coefficient of the chamber after closing ($k_{c,2}$) and the stiffness coefficient of the skin ($k_s$ = 1.765 N/mm) (Supplementary Fig. 8a and Equation (18)). The stiffness of skin is calculated by substituting Young's modulus of skin ($E_s$ = 100 kPa), thickness (t = 1.5 to 2.5 mm), outer radius ($r_2$ = 5 mm), and inner radius ($r_1$ = 3.5 mm) into Equation (19), resulting in a skin stiffness of $k_s$ = 1.6 to 2.7 N/mm. It was confirmed that the breathable chamber have the suitable variable stiffness for the design criteria.

$$k_c = \begin{cases} k_{c,1} & , \delta_c \leq 1\,mm \\ k_{c,2} & , \delta_c > 1\,mm \end{cases} \tag{13}$$

$$\delta_b = \delta_c + \delta_s = F_c/k_c + F_s/k_s \tag{14}$$

$$(+)\uparrow \sum \vec{F} = -F_b + F_c = 0 \tag{15}$$

$$\therefore F_b = F_c \tag{16}$$

$$\delta_b = \delta_c + \delta_s = F_b\left(\frac{1}{k_c} + \frac{1}{k_s}\right) \tag{17}$$

$$k_{c,1} \ll k_{c,2}\, , k_s \tag{18}$$

$$k_s = \frac{E_s A}{t} \tag{19}$$

*Supplementary Note 4. SMA stress modelling*

To prevent permanent deformation or damage due to the cyclic actuation of the SMA wire, it is essential to consider the stress ( $\sigma$ ) exerted on the SMA wire due to the critical force applied by the bistable beam. The stress applied to the SMA wire should be designed not to exceed the material's yield strength. One method to reduce SMA stress is to distribute the tension by using multiple wires instead of a single wire. Another method is to increase the wire's diameter, thereby reducing stress. To calculate the stress applied to the wire, a free body diagram consisting of the critical force ($F_{beam}$) and tension ($T_{SMA}$) applied to the SMA can be represented as shown in Supplementary Fig. 10. The tension according to the characteristics of the SMA wire is as follows,

$$\sigma = \frac{nF_{beam}}{d^2 \pi \sin \theta}$$

where n is the number of rows of SMA wire, d is the diameter of wire.

**Reference**


1 Imhof, B., Xiao, P. & Angelova-Fischer, I. in *Non invasive diagnostic techniques in clinical dermatology* 345-352 (Springer, 2013).

2 Tagami, H., Kobayashi, H. & Kikuchi, K. A portable device using a closed chamber system for measuring transepidermal water loss: comparison with the conventional method. *Skin Research and Technology: Official Journal of International Society for Bioengineering and the Skin (ISBS)[and] International Society for Digital Imaging of Skin (ISDIS)[and] International Society for Skin Imaging (ISSI)* **8**, 7-12 (2002).

3 Sim, D., Kim, S. M., Kim, S. S. & Doh, I. Portable skin analyzers with simultaneous measurements of transepidermal water loss, skin conductance and skin hardness. *Sensors* **19**, 3857 (2019).

4 Imhof, R., De Jesus, M., Xiao, P., Ciortea, L. & Berg, E. Closed-chamber transepidermal water loss measurement: microclimate, calibration and performance. *International journal of cosmetic science* **31**, 97-118 (2009).

5 Nuutinen, J. *et al.* A closed unventilated chamber for the measurement of transepidermal water loss. *Skin Research and Technology* **9**, 85-89 (2003).
