# Supplementary material for: Breathable, wearable skin analyzer for reliable long-term monitoring of skin barrier function and individual environmental health impacts
Source: Nat Commun. 2025 Oct 15;16:9149. doi: 10.1038/s41467-025-64207-2 (PMC12528383; doi:10.1038/s41467-025-64207-2)
Supplement: Supplementary file 2 — Description of Additional Supplementary Files [file 41467_2025_64207_MOESM2_ESM.pdf]

Description of Additional Supplementary Files

Supplementary Movie 1. Bistable actuating based on SMA for opening and closing the breathable chamber

Supplementary Movie 2. Demonstration of the wireless skin health analyzer for reliable long-term monitoring
